# Effects of Impeller Geometry on the 11α-Hydroxylation of Canrenone in Rushton Turbine-Stirred Tanks

**DOI:** 10.4014/jmb.2104.04002

**Published:** 2021-05-19

**Authors:** Shaofeng Rong, Xiaoqing Tang, Shimin Guan, Botao Zhang, Qianqian Li, Baoguo Cai, Juan Huang

**Affiliations:** 1 Department of Biological Engineering, Shanghai Institute of Technology, Shanghai 201418, P.R. China; 2 Department of Biological Engineering, East China University of Science and Technology, Shanghai 200237, P.R. China

**Keywords:** 11α-Hydroxycanrenone, impeller geometry, fungal metabolism, CFD, fluid velocity, shear strain rate

## Abstract

The 11α-hydroxylation of canrenone can be catalyzed by **Aspergillus* ochraceus* in bioreactors, where the geometry of the impeller greatly influences the biotransformation. In this study, the effects of the blade number and impeller diameter of a Rushton turbine on the 11α-hydroxylation of canrenone were considered. The results of fermentation experiments using a 50 mm four-blade impeller showed that 3.40% and 11.43% increases in the conversion ratio were achieved by increasing the blade number and impeller diameter, respectively. However, with an impeller diameter of 60 mm, the conversion ratio with a six-blade impeller was 14.42% lower than that with a four-blade impeller. Data from cold model experiments with a large-diameter six-blade impeller indicated that the serious leakage of inclusions and a 22.08% enzyme activity retention led to a low conversion ratio. Numerical simulations suggested that there was good gas distribution and high fluid flow velocity when the fluid was stirred by large-diameter impellers, resulting in a high dissolved oxygen content and good bulk circulation, which positively affected hyphal growth and metabolism. However, a large-diameter six-blade impeller created overly high shear compared to a large-diameter four-blade impeller, thereby decreasing the conversion ratio. The average shear rates of the former and latter cases were 43.25 s^-1^ and 35.31 s^-1^, respectively. We therefore concluded that appropriate shear should be applied in the 11α-hydroxylation of canrenone. Overall, this study provides basic data for the scaled-up production of 11α-hydroxycanrenone.

## Introduction

The steroid compound 11α-hydroxycanrenone is an important intermediate for the synthesis of the antihypertensive drug eplerenone, which can be obtained by 11α-hydroxylation of canrenone [1]. Studies have revealed that biotransformation is a common method for the synthesis of steroid hormone drug intermediates [2, 3]. For example, *Aspergillus* spp. and *Rhizopus* spp. are frequently used for the 11α-hydroxylation of canrenone, in which cytochromes P450 (P450s) are the key to bioconversion [4, 5]. P450s belong to the group of external monooxygenases, and the supply of oxygen is the basic requirement for the 11α-hydroxylation of canrenone [6].

It is known that aerobic fermentation is affected by the type and geometry of the impeller in stirred tanks [7-9]. The gas dispersion, flow pattern and mixing in bioreactors are all affected by these factors. The gas distribution has a great influence on the dissolved oxygen (DO) content in fermentation broth [10, 11]. Shin *et al*. indicated that the oxygen supply of fermentation broth, which was the restrictive factor for enzyme production by *Aspergillus oryzae*, was affected by impeller geometries [12]. The growth of microorganisms and the expression of enzymes in fermentation were also affected by DO [13, 14]. Regarding the flow pattern and mixing, it is known that in submerged fermentation, the product quality is affected by these factors [15, 16]. Li *et al*. found that mixing was improved by increasing the impeller diameter by approximately 10% in the enzyme production of *A. oryzae* fermentation [17]. In the nemadectin fermentation process, Wang *et al*. found that the radial-axial impeller combination with a time average velocity of 0.38-0.54 U_tip_ was more beneficial for the activity of *Streptomyces cyaneogriseus* ssp. noncyanogenus and nemadectin biosynthesis than other combinations[18].

In addition, the properties of the fermentation broth and microbial morphology, which greatly influence fermentation, are affected by the impeller type [19, 20]. Li *et al*. showed that the hyphal morphology was related to the hydrodynamic stress and hyphal tensile strength [21]. There is also a correlation between the hydrodynamic stresses and impeller types. Ghobadi *et al*. increased the pellet porosity during mixing via a flexible shaft agitator, resulting in an increase in oxygen mass transfer and average shear stress, which decreased the viscosity and the rheological index of the cultivation broth. In this way, the α-amylase activity of *A. oryzae* was improved [22]. Jüsten *et al*. observed that changes in the morphology and specific penicillin production rate of *Penicillium chrysogenum* depended on the impeller geometry at a given power per unit volume of liquid (P/V). The highest production using the Rushton turbine was achieved at the lowest speed due to the suitable shearing environment [23]. Thus, the range of shear stress should be determined according to the specific situation in fungal fermentation.

The hydrodynamics in bioreactors can be better analyzed using computational fluid dynamics (CFD) [24-26]. In the study of Amer *et al*., CFD was used to simulate gas-liquid mixing in a stirred tank. The oxygen mass transfer coefficient was correlated with geometrical parameters, requiring relatively less time and fewer experiments [27]. In a study by Duan *et al*., a CFD model was used to investigate the influence of different impeller combinations on fluid dynamics in a 7-L bioreactor for the fermentation of cephalosporin C (CPC), in which an impeller combination with a higher oxygen transfer coefficient and moderate shear force was the prerequisite for efficient CPC production[28]. Xia *et al*. used CFD to study the fluid dynamics of three impeller combinations and its effects on the physiology of *Streptomyces avermitilis* used for avermectin fermentation, which showed obvious advantages in determining the main factors affecting the fermentation, mixing efficiency and shear environment [29]. All of these studies fully demonstrate the reliability of numerical simulations in the study of flow field characteristics in stirred bioreactors.

As mentioned above, the outcome of biotransformation is closely related to the impeller type in the bioreactor. Therefore, the geometric parameters of impellers are crucial to biotransformation. However, there are few studies on the effect of impeller design on the production of 11α-hydroxycanrenone. Contente *et al*. only developed a high-yielding biological process of 11α-hydroxycanrenone under the conditions of oxygen-enriched air supply [30]. Thus, in the present work, the effects of the impeller geometry of Rushton impellers on the biotransformation of 11α-hydroxycanrenone were studied both experimentally and through numerical simulation. We examined the influence of the blade number and impeller diameter and believe that this research well elucidates the effects of impeller geometry on steroid biosynthesis.

## Materials and Methods

### Bioreactor and Impellers

**Fig. 1:**
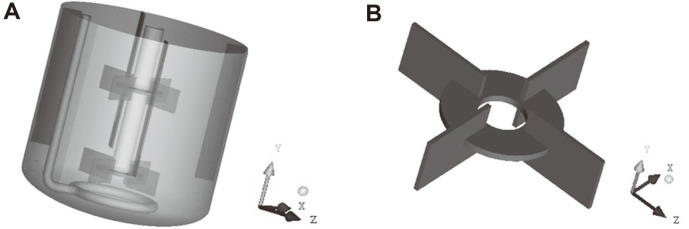
Geometry of (A) bioreactor and (B) impeller.

The experiments were performed in elliptical-bottomed cylindrical, 1,000 ml, stirred-tank bioreactors with a liquid volume of 700 ml and a liquid height of 102 mm. The experimental device is shown in Fig. 1A, in which the diameter of the tank is 100 mm, and four baffles are evenly distributed in the stirred tank that are 10 mm wide and 165 mm long. On the circular distributor with a diameter of 48 mm, twenty holes venting to the bottom of the tank are evenly distributed. Four kinds of Rushton turbine impellers were employed: a 50 mm four-blade impeller (1-1), 50 mm six-blade impeller (1-2), 60 mm four-blade impeller (2-1) and 60-mm six-blade impeller (2-2). The geometry of each impeller is shown in Fig. 1B, and the dimensions of the impellers are shown in Table 1.

### Materials and Reagents

Canrenone (≥ 98% purity) was purchased from Shanghai Yuanye Bio-Technology Co., Ltd., China. All chemicals and reagents used were of analytical grade or higher.

### Microorganism Cultivation and Biotransformation Experiments

The strain *A. ochraceus* MF010 was used in this study. The spores scraped from slant medium were inoculated into 50 ml seed medium in a 250 ml flask. The seed medium contained 10 g/l glucose, 20 g/l corn steep liquor, and 2.0 g/l (NH_4_)_2_SO_4_, and the pH was adjusted to 6.4. After cultivation at 28°C and 180 rpm for 18 h, 70 ml seed medium was used to inoculate 630 ml fermentation medium in the 1,000 ml bioreactor. The pH of the fermentation medium containing 25 g/l glucose, 20 g/l soy peptone, and 2.0 g/l (NH_4_)_2_SO_4_ was adjusted to 4.4. After 18 h of incubation at 28°C and 180 rpm, 10 g/l canrenone was added to the bioreactor to prepare for the biotransformation. The aeration rate and agitation speed procedures are shown in Table 2. In addition, the total conversion time is 60 h.

### Cold Model Experiments

The hyphae were cultured for 48 h and washed with phosphate-buffered saline (PBS) three times and then collected. Equal weight hyphae were stirred at 500 rpm and 2.0 vvm for 12 h in four kinds of tanks containing 700 ml PBS, and 2.5 g/l amino acids were added to prevent premature autolysis. The contents of protein and amino acids were detected to characterize the permeability of hyphae. The hyphae were stirred for 12 h in four bioreactors and then washed with PBS three times and collected again. Equal weight hyphae were cultured at 200 rpm and 28°C for 6 h in 250 ml flasks containing 50 ml PBS, 2.5 g/l glucose and 2 g/l canrenone, and conversion of 11α-hydroxycanrenone was measured by high-performance liquid chromatography (HPLC). The conversion ratio of 11α-hydroxycanrenone with the hyphae collected in the shake flask cultured at 200 rpm and 28°C for 12 h was taken as the control to calculate the enzyme activity retention (EAR) values according to the following formula:

$$\text{EAR(\%) = }\frac{\text{Conversion ratio of hyphae collected in bioeactor}}{\text{Conversion ratio of hyphae collected in flask}}\times 100\%.$$

### Analytical Methods

**Analysis of the conversion ratio.** HPLC (Agilent 1260, Agilent Technologies, Inc., USA) samples were extracted by ethyl acetate and filtered through a 0.22 μm filter. The column (Agilent 5 HC-C18 250 × 4.6 mm) temperature was 30°C, and the mobile phase was a mixture (v:v, 8:2) of methanol and doubly distilled water (ddH_2_O) with a flow rate of 0.8 ml/min at a detection wavelength of 280 nm. The concentration of canrenone in the aqueous phase was determined using the same chromatographic conditions after the fermentation broth was filtered through a 0.22 μm filter. The consumption rate of canrenone per 12 h was calculated according to the following formula:

$$\text{Consumption rate of canrenone (mg/g) = }\frac{10\times (\text{Conversion ratio after 12h - Conversion ratio before 12 h) }\times \text{ 1000}}{0.5\times (\text{Dry biomass weight after 12 h + Dry biomass weight before 12 h)}}.$$

**Determination of the DO, pH and viscosity.** The DO and pH of fermentation broth were measured using the bioreactor's self-equipped DO electrode and pH electrode. An SNB-1 digital viscometer (Shanghai Precision and Scientific Instrument Co., Ltd., China) was used to measure the apparent viscosity of fermentation broth.

**Characterization of microbial activity.** The fermentation broth was centrifuged for 5 min at 5,000 ×*g*. The centrifugal precipitate was washed with ddH_2_O three times, and the biomass concentration was calculated by the dry weight of the hyphae dried at 105°C. The supernatant of fermentation broth after centrifugation was used for reduced sugar concentration measurements by the 3,5-dinitrosalicylic acid (DNS) method [31]. The morphology of the hyphae washed with ddH_2_O was observed by scanning electron microscopy (SEM, FEI Quanta200 FEG, Netherlands) at an accelerating voltage of 5.00 kV. Samples were freeze-dried and coated with gold under high-vacuum conditions.

**Measurement of hyphal inclusions.** The leakage of protein and changes in amino acids were used as evaluation indicators for hyphal permeability. Under cold-mode conditions, the supernatant was taken after the culture was centrifuged (8,000 ×*g*, 5 min). The protein content was determined by the Coomassie brilliant blue method [32], and amino acids were determined by the ninhydrin method [33].

### Numerical Simulation Methods

ANSYS ICEM CFD 16.0 (ANSYS Inc., USA) was used to generate the mesh of the bioreactor model. The bioreactor was divided into three parts: a tank part including a tank shell with four baffles and two stirring parts. An unstructured mesh was adopted, in which the elements of the entire model were tetrahedrons, and the quality of all the grids was larger than 0.3.

Ansys CFX 16.0 (ANSYS Inc.) was used for the simulation of the fluid dynamics in the stirred tank. The simulation conditions were set according to the fermentation conditions of 48 h. The tank part was set to the stationary domain, and the two stirring parts were set to the rotating domain at a speed of 500 rpm. The gas phase and liquid phase were both created in all domains. The gas phase was set as air at 25°C and dispersed fluid. The liquid phase was defined as a non-Newtonian fluid, and the rheological equation was μ_a_=7.0356 × $\overset{.}{\gamma }$^-0.839^, where μ_a_ was the apparent viscosity (Pa·s) and $\overset{.}{\gamma }$was the shear rate (s^-1^). A standard k-ε turbulence model was employed to simulate gas-liquid turbulent flow in the stirred bioreactors. The broth surface was created as a degassing outlet to represent the free surface where the dispersed phase (air at 25°C) escaped, and the continuous phase (non-Newtonian fluid) saw this boundary as a free-slip wall and did not leave the domain. The other surfaces were created as the boundary types of walls, where the dispersed phase saw those boundaries as free-slip walls and the continuous phase saw those boundaries as no-slip walls. The stirring shaft was set to a rotating wall with a speed of 500 rpm in the stationary domain. High resolution was used as the advection scheme when setting the solver control. The maximum iteration was 100 in convergence control, and the convergence criteria value was 10^-4^, which meant that the solving procedure would terminate when the root mean square (RMS) values of all variables were smaller than 10^-4^.

## Results

### Bioconversion of Canrenone

**Fig. 2:**
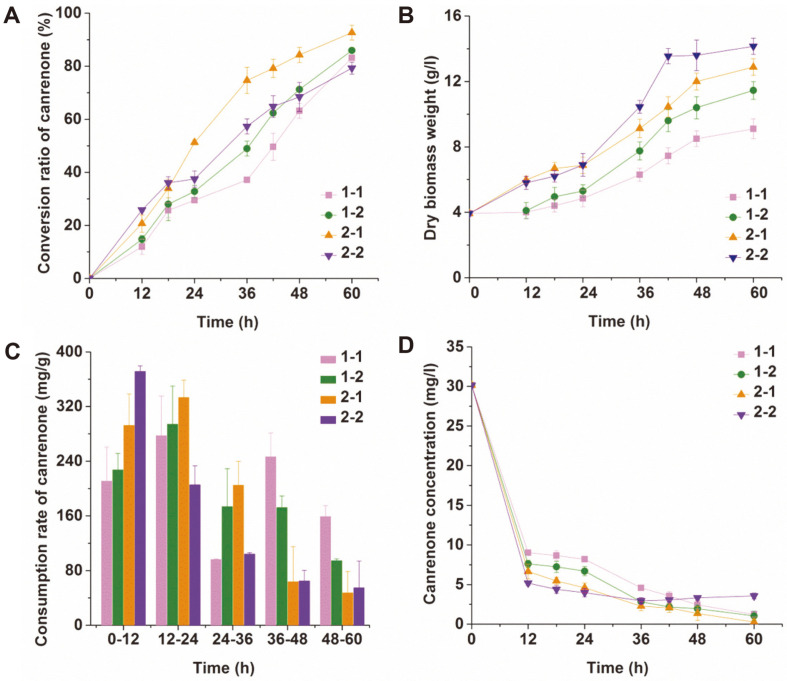
Effects of impeller geometry on (A) canrenone conversion, (B) dry biomass weight, (C) canrenone consumption and (D) canrenone concentration in aqueous phase.

The effects of blade number and impeller diameter on the biotransformation when using the Rushton turbine were compared. Fig. 2A shows that the conversion ratio under agitation with 1-2 was 3.40% higher than that with 1-1. A conversation ratio of 92.65% was obtained under agitation with 2-1, which was 11.43% higher than that with 1-1. We concluded that the conversion ratio of canrenone can be increased by increasing both the blade number and impeller diameter on the basis of a 50 mm four-blade impeller. The latter was more effective than the former. However, with the agitation of a large-diameter or six-blade impeller, increases in the blade number or the diameter will lead to a decrease in the conversion ratio. Fig. 2A shows that the conversion ratio under agitation with 2-2 was 14.42% lower than that with 2-1. The lowest conversion ratio of 79.29% was obtained from the reactor stirred by the impeller with a large diameter and six blades.

According to Fig. 2B, the biomass concentration mixed with 2-2 was the highest, although the conversion ratio under this condition was the lowest. The results showed that the dry biomass weights were 9.10, 11.45, 12.88, and 14.15 g/l at 60 h when stirred by 1-1, 1-2, 2-1, and 2-2, respectively. Increasing the blade number and impeller diameter can both accelerate microbial growth. Compared to the other three kinds of impellers, 2-2 was beneficial to microbial growth. It is speculated that the hyphal concentration was not the only factor affecting the conversion ratio in the 11α-hydroxylation of canrenone.

To elucidate the above results, the canrenone consumption rate per mass biomass over a time interval of 12 h is shown in Fig. 2C. It can be seen from the figure that a low consumption rate of canrenone was obtained when the broth was stirred by 2-2 within 12-60 h, although the canrenone consumption rate was the highest within 0-12 h. The low utilization rate at late biotransformation caused a decrease in the conversion ratio. It is speculated that the decrease in the utilization rate is caused by a decrease in biological activity.

It is known that the solubility of canrenone affects the rate of 11α-hydroxylation. The concentration of canrenone in the aqueous phase shown in Fig. 2D gradually decreased with the 11α-hydroxylation of canrenone. After 36 h of biotransformation, the concentration of canrenone in the aqueous phase with 2-2 increased gradually, and the final detection concentration was 3.56 mg/l, which was 191.80% higher than that with 1-1. This result indicated that the utilization rate of canrenone was reduced in the late conversion stage when stirred by 2-2, which is consistent with the canrenone consumption rate mentioned above.

The above results show that the impeller geometry may influence the microbial viability, so the metabolic activity and hyphal morphology of *A. ochraceus* were measured and described below.

### Variation in the Physicochemical Properties of Fermentation Broth

**Fig. 3:**
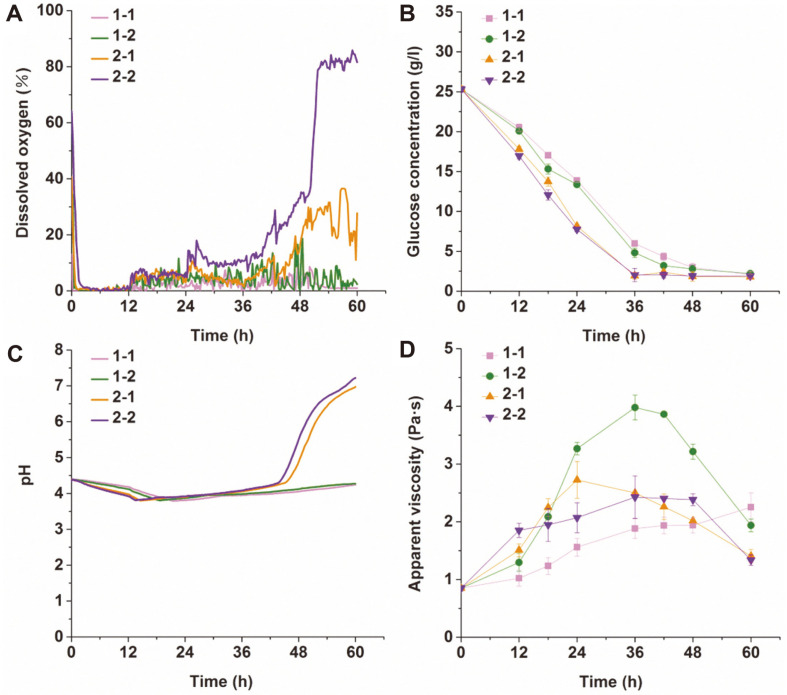
Effects of impeller geometry on (A) dissolved oxygen, (B) glucose concentration, (C) pH and (D) apparent viscosity of fermentation broth.

The DO value, reducing sugar content, pH and apparent viscosity of fermentation broth were evaluated to characterize the biological activity. The results are shown in Fig. 3.

The results shown in Fig. 3A illustrated that the geometric parameters of the impeller affected the DO content of fermentation broth. The time average DO values with 1-1, 1-2, 2-1, and 2-2 were 2.14%, 3.76%, 9.26%, and 22.31%, respectively, within 60 h. As Fig. 3 indicates, the impeller with more blades and a large diameter was conducive to high DO values in fermentation broth. The DO values obtained from the agitation of 60 mm impellers (2-1 and 2-2) were significantly higher than those with 50 mm impellers (1-1 and 1-2). After 48 h of biotransformation, the DO level increased rapidly when mixing with 2-2. Although the biomass concentration was the highest when stirred by 2-2, the conversion ratio decreased due to the decrease in microbial metabolic activity in the late stage of the biotransformation.

From Fig. 3B, it was observed that increasing the blade number and impeller diameter enhanced the metabolism of reducing sugars. There was a reduced metabolic rate of reducing sugars mixed by 2-2 after 24 h, which indicated that the metabolic activity of hyphae was significantly reduced. Various organic acids are produced when reducing sugars are utilized rapidly, which decreases the pH of the fermentation broth; the variation in pH shown in Fig. 3C confirmed this occurrence. When the ratio of carbon to nitrogen in the fermentation broth decreased, nitrogen sources were metabolized rapidly, and the pH of the system began to increase. In addition to nitrogen source metabolism, the ammonia released by hyphal autolysis caused an increase in pH. When agitating with 2-1 and 2-2, the pH of the culture increased sharply at 44.5 h and 43.0 h, respectively, which indicated that impellers with large diameters accelerated hyphal autolysis.

In terms of the apparent viscosity of the cultures during biotransformation, we found that there was a great difference when various impellers were employed (Fig. 3D). The apparent viscosity of the culture gradually increased when 1-1 was used within 60 h. For agitation with 1-2, 2-1 and 2-2, the apparent viscosities all showed a downward trend after increasing. We also found that the apparent viscosity was the lowest (1.334 Pa·s) when stirred by 2-2 at 60 h, although the hyphal concentration was the highest compared with the other three kinds of impellers. The results signified that the biomass concentration was not the only factor affecting the apparent viscosity of the fermentation broth.

The results shown in Fig. 3 indicated that the geometric parameters of impellers created significant differences in the properties of fermentation broth. Rapid increases in the pH and DO content with 2-2 were observed at the end of conversion. The autolysis of filamentous fungi will cause an increase in pH, a reduction in the oxygen utilization rate and a decrease in the apparent viscosity of broth [34]. Therefore, it is reasonable to speculate that the damage to fungal activity with 2-2 resulted in the low biotransformation rate observed in the late stage of biotransformation. This indicates that the transformation activity of *A. ochraceus* was affected by the geometric parameters of the impellers.

During autolysis, the morphology of the hyphae changes, and intracellular substances are released at the same time. Thus, the hyphal morphology and leaked substances were investigated to confirm that the 11α-hydroxylation activity of *A. ochraceus* was affected by the geometry of the impellers.

### Hyphal Morphology

**Fig. 4:**
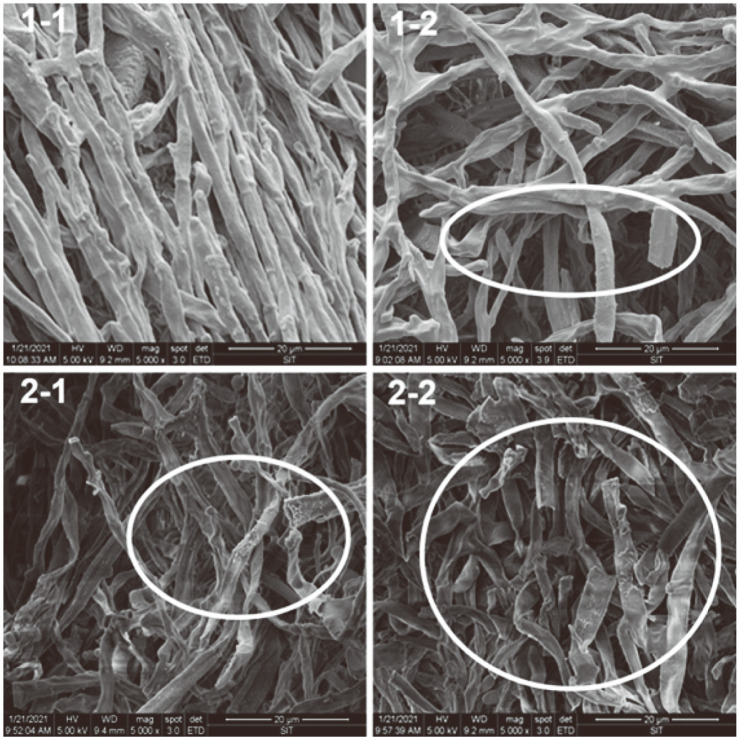
SEM images of hyphal morphology. Hyphae was collected and observed at biotransformation of 48 h.

Fig. 4 shows SEM images of hyphae stirred by four impellers. The hyphae after agitation by 1-1 are smooth and thick. By contrast, the hyphal surface is rough and the thickness is not uniform when stirred by 1-2 and 2-1, in which the lack of uniformity with 2-1 is more serious than that with 2-2. The hyphae were broken most seriously, with thinner hyphae and fewer branches, when the bioreactor was equipped with 2-2. This result showed that increasing the blade number and impeller diameter can accelerate the autolysis of hyphae, where the influence of the latter was more significant than that of the former. The apparent viscosity of fermentation broth decreased sharply due to the severely broken hyphae after 36 h when using 2-2. These results were consistent with the report that the apparent viscosity is related to the concentration and morphology of microorganisms [35].

### Leakage of Hyphal Inclusions in the Cold Model Experiment

**Fig. 5:**
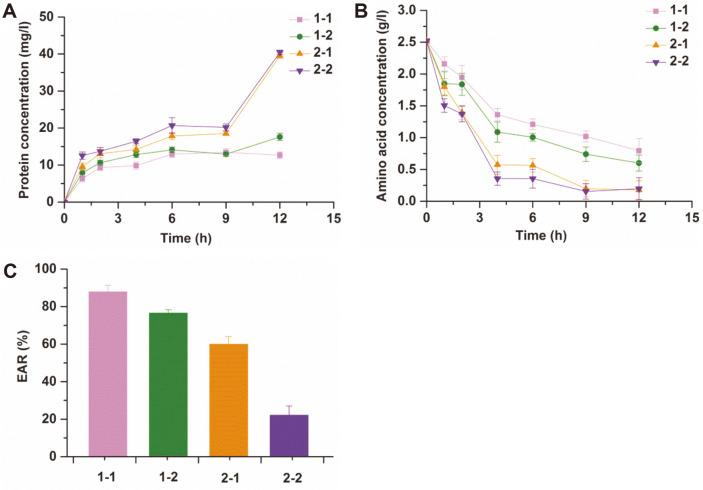
Effects of impeller geometry on (A) leaky protein, (B) amino acid concentration and (C) EMR of *A. ochraceus*.

To further investigate the effect of impeller geometry on the activity of *A. ochraceus*, changes in the protein and amino acid concentrations in the cold model system were performed. At the same agitation speed, it is obvious that 1-1 had the lowest content of protein leakage and that 2-2 had the highest, according to the results shown in Fig. 5A. The protein content increased sharply from 9 h when impellers of 60 mm diameter were used, which indicated that increasing the impeller diameter accelerated protein leakage. Fig. 5B shows that the metabolic rates of amino acids were different using the four agitators. The concentration of amino acids in the system stirred by 2-2 showed an increasing trend from 9 h after the decrease, which means that the permeability of the hyphae was obviously increased.

According to the results of protein and amino acids concentration, we concluded that a large-diameter impeller accelerated the release of hyphal inclusions. In this case, the EAR value of filamentous fungi was calculated, as given in Fig. 5C. There were significant differences between the EAR values of different impeller stirrers, which indicated that mechanical agitation had an effect on the hydroxylation activity of fungi. The EAR values of 1-2, 2-1, and 2-2 were 12.93%, 31.75%, and 74.86% lower than that of 1-1, respectively, which indicated that the decreases in the 11α-hydroxylation activity of *A. ochraceus* were related to the increases in the blade number and diameter of Rushton turbine impellers.

The above results show that the DO content, microbial metabolism, hyphal morphology and biotransformation activity were all affected by the geometric parameters of the impellers, which in turn influenced the conversation ratio of canrenone. The variation in the properties of the broth is caused by the hydrodynamics in the stirred tank. Therefore, the hydrodynamics were investigated in bioreactors equipped with the four kinds of impellers using numerical simulation.

### Numerical Simulation Results

**Fig. 6:**
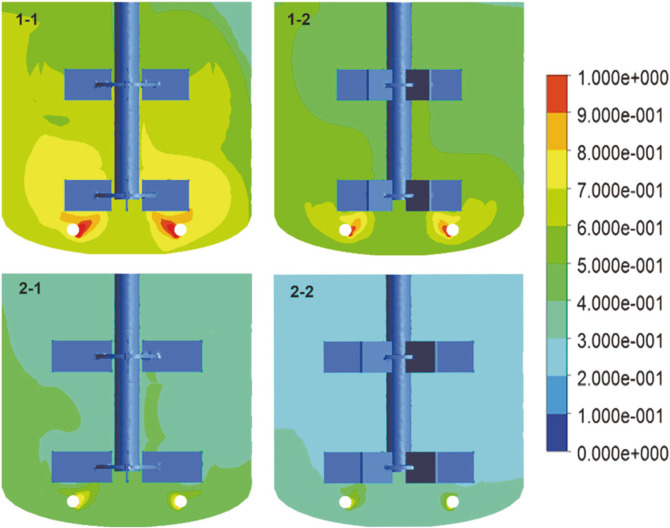
Effects of impeller geometry on air volume fraction.

**Gas holdup.** The distribution of the gas on the mid-plane of the bioreactor is shown in Fig. 6. The gas holdup in the lower region of the tank is higher than that in the upper region. This condition was created by the downward discharge flow in the lower circulation area, prolonging the residence time of the bubbles. It was obvious that the gas distribution of the six-blade impeller and 60 mm diameter impeller on the mid-plane was more uniform than that of the four-blade impeller and 50 mm diameter impeller. An impeller with a large diameter and more blades was effective for gas dispersion.

**Fig. 7:**
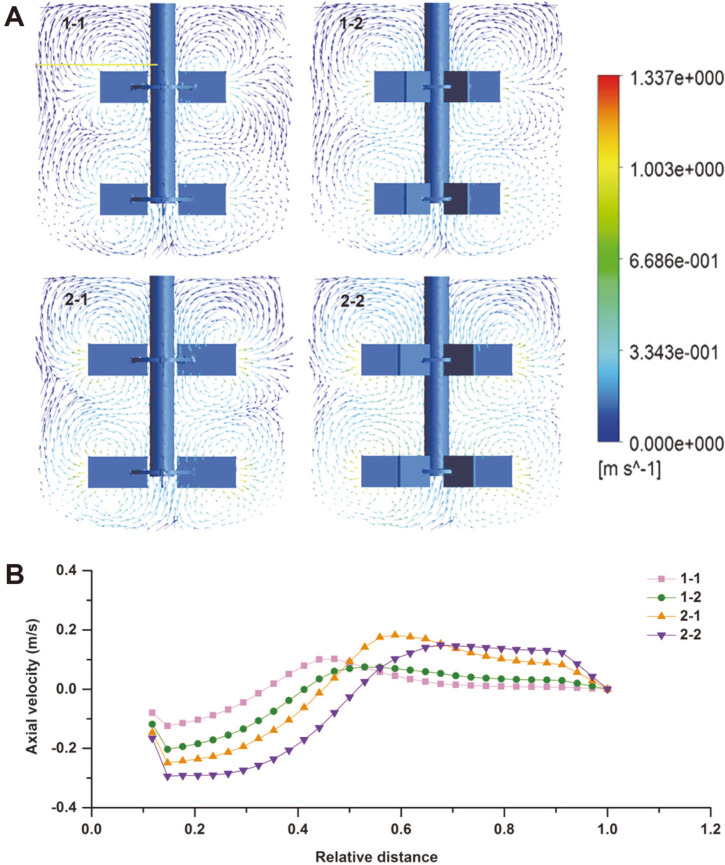
Effects of impeller geometry on (A) flow pattern, (B) radial velocity and (C) axial velocity of the fluid. The position of velocity generation is located on the yellow horizontal line in Fig. 7A, extending from the center of the stirring shaft to the tank wall.

**Fluid pattern.** Fig. 7A shows the flow pattern at the mid-plane of the bioreactors. Under the stirring of double Rushton impellers, the fluid was discharged from the impeller tip toward the tank wall. As the fluid hit the wall, it was divided into two loops circulating at the top and the bottom of the blade, with obvious radial flow. The fluid circulations do not interact with each other when stirred by 1-1, and the proportion of the high velocity region was the smallest and only appeared near the blade. When the blade number increased to six (1-2), the fluid circulation began to contact. When the diameter of the impeller increased to 60 mm (2-1 and 2-2), the fluid circulation areas expanded to the tank wall and crossed. The flow pattern of 2-2 was more regular than that of 2-1. At the same time, the high-speed fluid region around the impeller further expanded. The mixing dead zone was decreased, especially the region between the upper and lower impellers and at the tank wall. A Rushton impeller with a large diameter and more blades was beneficial to fluid mixing.

The axial velocity along the radial positions of the tank near the blade is shown in Fig. 7B. This figure shows that the axial velocity of the fluid is highest when stirred by Rushton impellers with more blades and large diameters. The circulation area of the flow field mixed with 60 mm impellers is significantly wider than that of the 50 mm impellers. In this case, the width of the circulation area using 2-2 was approximately 1/2 of the tank diameter, which was the largest in this study.

**Fig. 8:**
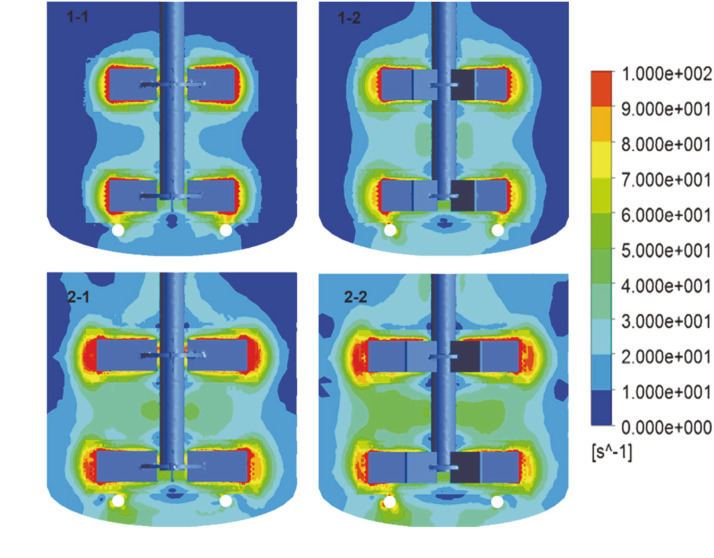
Effects of impeller geometry on shear strain rate.

**Shear strain rate and stirring power.** Hydrodynamic shear was produced when the broth was agitated. Fig. 8 shows the shear strain rate distribution of the fluid stirred by different impellers. The results indicated that the shear strain rate in the blade region was higher than that in the other regions in the bioreactor, and it decreased with increasing distance to the impeller. Compared with the 50 mm diameter impeller, the shear strain rate increased at the region of the liquid surface and the tank wall when equipped with a 60 mm diameter impeller in the bioreactors. When using 2-2, the low shear area only appeared at the top of the mid-plane of the bioreactor.

The average shear strain rate and specific stirring power are given in Table 3. The average shear strain rate of the fluid was 19.36 s^-1^, and the P/V was 1.46 kW/m^3^ when 1-1 was used. For 1-2, 2-1, and 2-2, the average shear strain rates were 23.53%, 120.59%, and 192.16% higher than that of 1-1, respectively; the P/V values were 29.34%, 82.39%, and 123.40% higher than that of 1-1, respectively. These results showed that increasing the blade number and impeller diameter increased the shear strain rate and the specific power of the impeller. The effect of the impeller diameter was more obvious than that of the blade number.

## Discussion

The bioconversion ratio of canrenone is determined by various factors including the DO content, the metabolism of *A. ochraceus*, and the mixing and the shear stress of the fluid. All of these factors are influenced by the geometry of the impellers.

The biochemical reaction rate of *A. ochraceus* was affected by fluid mixing in fermentation broth. Mixing enhances the mass and heat transfer and suspends the microorganisms and substrate in the system, promoting the reaction [36, 37]. According to the results of fluid flow when stirred by different impellers studied in this article, increasing the blade number and impeller diameter improved the mixing in the tank, especially the axial flow velocity. The maximum axial velocities at agitation settings of 2-1 and 2-2 with a 60 mm diameter were 0.25 m/s and 0.29 m/s in the upper blade area, respectively, while those of 1-1 and 1-2 were 0.12 m/s and 0.20 m/s, respectively. The axial velocity of fluid is the key factor in mixing, and large-scale impellers are used to improve the mixing of the fluid [38, 39]. The circulation was better using impellers with large diameters because of the increase in blade tip linear velocity and the decrease in fluid apparent viscosity. In the mixed region of high fluid velocity, the gas was dispersed more evenly and DO content increased.

Oxygen needs to be dispersed and dissolved in the aqueous phase to be used by microbial cells in the process of oxygen consumption biotransformation [40, 41]. Therefore, it is essential to maintain the supply and delivery of oxygen for transformation. According to the gas holdup distribution in the bioreactor, increasing the blade number and impeller diameter can improve both the uniformity of gas in the fluid and increase the DO content. The results showed that 332.71% and 493.35% increases in the time average DO content were achieved by increasing the impeller diameter by 20% when mixing with the four-blade impeller and six-blade impeller, respectively. High power input is beneficial for air dispersion [42]. The simulation results showed that the stirring power of the Rushton impeller was higher with more blades or large diameters. It is easy for air to reach the state of complete dispersion in high-speed mixing fluid, which enables gas recycling [43]. At the same time, the apparent viscosity of fermentation broth decreases with increasing shear strain rate in the high-speed mixing area [44]. All these conditions are effective for increasing the gas-liquid contact area, reducing the gas transfer resistance and increasing the DO content in liquid phase [45]. Thus, the fermentation broth mixed with 2-1 or 2-2 had a high DO content. On the one hand, the high DO content accelerates the aerobic metabolism of reducing sugar, which provides the sufficient energy and carbon skeleton for the growth of microorganisms [46]. A high DO content is conducive to the enzyme expression during hydroxylation [47-49]. Under the condition of vigorous growth of *A. ochraceus*, the high biomass concentration provided more P450s than the low biomass concentration, which increased the 11α-hydroxylation rates of canrenone. On the other hand, oxygen is not only a necessity for the growth of microorganisms but also one of the substrates of 11α-hydroxylation [6]. When the large-diameter impellers were used, the fermentation broth with high DO content provided sufficient oxygen source for 11α-hydroxylation, which was beneficial for the biosynthesis of 11α-hydroxycanrenone. Therefore, it can be inferred that in the early stage of biotransformation, the high DO content in the medium is the main factor in the increased conversion ratio.

Compared with the other three kinds of impellers, we found that the dry biomass concentration from mixing by 2-2 was the highest at 60 h, which was 55.49% higher than that mixed by 1-1. However, the conversion ratio of 2-2 with the highest biomass was 79.29%, which was the lowest in this experiment. Therefore, the detailed influence of impeller geometry on microbial metabolism was explored further.

According to the physicochemical properties of the broth and SEM results regarding hyphal morphology, we concluded that increasing the blade number and impeller diameter of the Rushton turbine accelerated hyphal autolysis. The sharp increase of DO was related to the decrease of microbial activity. Within 36-60 h of biotransformation, the 11α-hydroxylation rates of canrenone under the agitation of the 50 mm impellers were significantly higher than that of the 60 mm impellers, even though the DO and biomass concentration of the former were lower than that of the latter. The yield of fermentation is affected by the hyphal morphology, which is greatly influenced by the shear in the tank [50, 51]. Filamentous fungal fermentation broth with differentiated and poor flexibility hyphae is more susceptible to shearing force because it is usually a non-Newtonian fluid [52]. The hyphae were damaged and autolysis was accelerated under the condition of continuous high shear stress, which was more obvious after the stable growth period. Autolysis was the most serious when agitation was provided by 2-2, resulting in severe damage to the hyphae. The average shear strain rate of 43.25 s^-1^ was beyond the tolerance range of the hyphae in the late stage of biotransformation under this condition, resulting in a significant decrease in the metabolic activity of *A. ochraceus* and the lowest conversion ratio (79.29%). Under the condition of a effective mixing and lower average shear strain rate (35.31 s^-1^) for the agitation with 2-1, the highest conversation ratio (92.65%) was achieved due to the lower autolysis and better morphology of the hyphae than that with 2-2.

In addition to the reduction in metabolic activity, it was speculated that the decreased hydroxylation capacity of *A. ochraceus* was related to the leakage of protein. The 11α-hydroxylase complex is very sensitive to shear, and more effective biotransformation is carried out with fluffy and young particles in the hydroxylation of steroids with filamentous fungi [53]. At the same time, the excommunicated proteins may play a key role in substance transport and energy conversion [54]. The EAR values of filamentous fungi with high inclusion leakage were low in the cold model experiment. Therefore, the conversion rate gradually decreased because of the intense fluid shear strain rate and serious leakage of inclusions when stirred by 2-2, although it had the highest conversion ratio at 0-18 h. As shown in Fig. 2C, the highest hydroxylation rate of canrenone was observed after 48 h when 1-1 was used because of the smallest shear strain rate (19.36 s^-1^). It can be concluded that it is vital to control the shear stress during the 11α-hydroxylation of canrenone.

In this study, the effects of the blade number and diameter of a Rushton impeller on the 11α-hydroxylation of canrenone were compared. We concluded that fluid flow and shear both impact the biological parameters and play an important role in the 11α-hydroxylation of canrenone. Increasing the blade number and the impeller diameter improved the fluid mixing in the stirred tank. The DO content of fermentation broth can be increased, and it had the positive effects on the microbial growth and 11α-hydroxylation of canrenone. At the same time, it was essential to apply an appropriate shear strain rate because high shear stress reduced the biotransformation activity. According to the characteristics of the 11α-hydroxylation of canrenone, it can be inferred that the increased conversion ratio of canrenone by the alteration of impellers was mainly because of the high DO content in the early stage and the good hyphal morphology in the late stage of bioconversion. In addition, both of the above conditions are beneficial for the activation of metabolism in *A. ochraceus*.

In summary, the fluid flow and shear in mixing fermentation broth must be controlled at the same time to ensure that the 11α-hydroxylation of canrenone can be carried out efficiently. Since reports about the influence of the impeller geometry on the biosynthesis of 11α-hydroxycanrenone have rarely been published, this research provides basic data for the industrial production of this compound.

## Figures and Tables

**Table 1:** Dimensions of four impellers.

Impeller	Blade number	Impeller diameter (mm)
1-1	4	50
1-2	6	50
2-1	4	60
2-2	6	60

**Table 2:** Operating conditions used for biotransformation experiments.

Conversion time (h)	Agitation speed (rpm)	Aeration rate (vvm)
0-12	350	1.5
12-24	450	2.0
24-36	500	2.5
36-60	500	2.5

^a^vvm: air volume/culture volume/minute.

**Table 3:** Effect of impeller geometry on power and average shear strain rate.

Impeller	Average shear strain rate (s^-1^)	P/V (kW/m^3^)
1-1	19.36	1.46
1-2	25.04	1.80
2-1	35.31	3.21
2-2	43.25	4.26
